# Competing length scales and 2D versus 3D dimensionality in relatively thick superconducting NbN films

**DOI:** 10.1038/s41598-023-46579-x

**Published:** 2023-11-09

**Authors:** Mikhail Belogolovskii, Magdaléna Poláčková, Elena Zhitlukhina, Branislav Grančič, Leonid Satrapinskyy, Maroš Gregor, Tomáš Plecenik

**Affiliations:** 1 Kyiv Academic University, Academician Vernadsky Blvd. 36, Kyiv, 03142 Ukraine; 2 Department of Experimental Physics, Faculty of Mathematics, Physics and Informatics, Comenius University Bratislava, 84248 Bratislava, Slovak Republic; 3 O.O. Galkin Donetsk Institute for Physics and Engineering, National Academy of Sciences of Ukraine, Nauki Ave. 46, Kyiv, 03028 Ukraine

**Keywords:** Materials science, Nanoscience and technology, Condensed-matter physics

## Abstract

Magneto-transport characteristics of 2D and 3D superconducting layers, in particular, temperature and angular dependences of the upper critical field *H*_c2_, are usually considered to be fundamentally different. In the work, using non-local resistance measurements at temperatures near the normal-to-superconducting transition, we probed an effective dimensionality of nm-thick NbN films. It was found that in relatively thick NbN layers, the thicknesses of which varied from 50 to 100 nm, the temperature effect on *H*_c2_ certainly pointed to the three-dimensionality of the samples, while the angular dependence of *H*_c2_ revealed behavior typical for 2D samples. The seeming contradiction is explained by an intriguing interplay of three length scales in the dimensionally confined superconducting films: the thickness, the Ginzburg–Landau coherence length, and the magnetic-field penetration depth. Our results provide new insights into the physics of superconducting films with an extremely large ratio of the London penetration depth to the Ginzburg–Landau coherence length exhibiting simultaneously 3D isotropic superconducting properties and the 2D transport regime.

## Introduction

In low-dimensional metallic layers, the motion of carriers is restricted in certain directions. Its effect on the charge transport in non-superconducting films is usually controlled by the interplay of *two* parameters, the sample thickness and the effective de Broglie wavelength of carriers. The dimensional reduction often results in significant changes of electronic structure and unexpected physical properties. This is also true for 2D superconductors such as metallic nm-thick layers, transition metal dichalcogenide films, some types of oxide interfaces and heterostructures, etc^1^. Many emerged properties as unexpected interface superconductivity^2^, a remarkable dome-shaped phase diagram^3^, high critical parameters^4^, superconductivity granularity^5^ as well as prospects for new practical applications^6^ have attracted great attention to 2D superconducting systems.

Let us, however, pay attention to the fact that, unlike normal metals, in superconducting layers there are not two but *three* characteristic length scales, the thickness (*d*), the coherence length (*ξ*), and the magnetic-field penetration depth (*λ*). Low-dimensional superconductivity is operationally defined as superconductivity in a system with dimension (s) comparable to or less than *ξ* and *λ*. In such specimens, temperature (*T*) and angular (*θ*) dependences of superconductor characteristics are indeed fundamentally different for 2D and 3D regimes. This is applied in particular to the upper critical field (*H*_c2_) completely destroying the superconducting state in a type-II superconductor, a key physical parameter knowing of which allows one to find out the *effective* dimension of a superconducting sample^7^. In the paper, we focus on magneto-transport measurements of NbN thin layers near the critical superconducting temperature (*T*_c_) whose specific features are determined by the temperature-dependent Ginzburg–Landau (GL) coherence length *ξ*(*T*) characterizing the distance over which superconductivity can vary without energy increase. While the GL approach is strictly valid only near *T*_c_, it has been extensively used to describe data over a much broader temperature range^7^, where its applicability cannot be justified. For this reason, we have restricted ourselves to a temperature interval 0.8*T*_c_ < *T* < *T*_c_ where the GL phenomenological approach can be definitely considered adequate.

As well known, type-II superconductors can retain zero-resistance state up to *H*_c2_ values by channeling the magnetic field through normal regions surrounded by circulating supercurrents shielding it within the cores (Abrikosov vortices). When thin superconducting films are in the mixed state, the fundamental difference between a 3D case and a 2D confinement lies in the distinct temperature dependence^7^. One can get an idea of the effective dimension of a superconducting film by comparing its thickness (*d*) with the GL out-of-plane coherence length *ξ*^⊥^(*T*). When *ξ*^⊥^(*T*) < *d*, then $\xi_{{}}^{ \bot } (T) = \xi_{0}^{ \bot } (d)/\sqrt {1 - T/T_{{\text{c}}} (d)}$ (3D case), otherwise, $\xi_{{}}^{ \bot } (T) = d$ (2D case). Hence, for an isotropic 3D sample with $\xi_{{}}^{ \bot } (T) = \xi_{{}}^{\parallel } (T) = \xi_{0} /\sqrt {1 - T/T_{{\text{c}}} }$,

1 $$\mu_{0} H_{{{\text{c2}}}}^{{}} (T) = \frac{{\Phi_{0} }}{{2\pi \xi_{0}^{2} }}\left( {1 - \frac{T}{{T_{{\text{c}}} }}} \right),$$

where *μ*_0_ is the vacuum magnetic permeability (magnetic constant), Φ_0_ is the magnetic flux quantum, $\xi_{0}$ is the zero-temperature coherence length. In an anisotropic 2D layer with $\xi_{{}}^{ \bot } (T) \ne \xi_{{}}^{\parallel } (T)$, the final relation depends on the magnetic field orientation. Namely, for out-of-plane and in-plane fields we get the following relations^8^

2 $$\mu_{0} H_{c2}^{ \bot } (T) = \frac{{\Phi_{0} }}{{2\pi \xi^{\parallel 2} (T)}} \sim (1 - T/T_{c} ),\;\;\;\;\;\;\;\;\mu_{0} H_{c2}^{\parallel } (T) = \frac{{\sqrt {12} \Phi_{0} }}{{2\pi \xi^{\parallel } (T)d}} \sim \sqrt {1 - T/T_{c} } .$$

If the film thickness *d* > *ξ*_0_, the transition from the root temperature dependence close to *T* = *T*_c_, where the GL coherence length diverges, to the linear one due to the temperature drop is just the crossover point *T* = T* from 2 to 3D behavior. It is the generally accepted way for analyzing changes in the effective dimensionality of thin superconducting films. Notice that the ratio *λ*/*ξ* in type-II superconductors exceeds $1/\sqrt 2$ according to the GL theory, hence, in most cases, at the crossing point T* where *d* = *ξ*^⊥^(T*) we have nevertheless an inequality *d* < *λ*(T*). Even more, in the case of a large excess of *λ* over *ξ*, there should be a wide range of thicknesses *d*, when a superconducting film is 3D in terms of the coherence length *ξ* and 2D in terms of the magnetic penetration depth *λ*. To identify such a state, we performed angle-dependent magneto-transport measurements. Another goal of the work was to separate the *anisotropic* orbital phenomenon arisen from the vortex penetration into a superconducting film discussed above from an *isotropic* paramagnetic response leading to the alternative Pauli-limited suppression of superconductivity.

The two tasks have been realized on thin layers of niobium nitride (NbN) known to be “the material of choice in developing future generation quantum devices”^9^ and offering a comprehensive platform for investigating the role of dimensionality in superconducting samples due to a huge excess of the value of *λ* over *ξ*.

Let us look at the related literature data. The magnetic penetration depth in NbN films is significantly higher than that in Nb where its value extrapolated to *T* = 0 K does not exceed 70 nm^10^. The scatter of the corresponding values for NbN layers is quite large, since they significantly depend on the method of the fabrication and the type of the substrate. At temperatures about 4 K, penetration depths for NbN films deposited epitaxially on sapphire or MgO at temperatures greater than 600 °C varied from 200 to 300 nm^11^ and between 280 and 600 nm for films deposited on Si or SiO^12,13^. The data for a wider range of substrates and buffer layers including oxidized Si, sapphire, and a variety of metal and metal nitride “seed” layers turned out to be scattered in the range 300–700 nm^14^. Nearly the same spread of *λ*(0) values was observed in ultrathin NbN layers^15^. The smallest *λ*(0) about 200 nm was obtained for epitaxial niobium nitride layers on MgO (100) substrates with the critical temperature of 16 K and the thickness about 100 nm^16^ and those on silicon substrate with *d* = 180 nm and *T*_c_ = 15.6 K^17^. As the NbN sample became thinner, this value increased up to 500 nm for thicknesses of the order of several nanometers^16^. There is more certainty for the coherence length value. The coherence length of the epitaxial NbN films on AlN-buffered c-plane Al_2_O_3_ was found to be *ξ*(0 K) = 2.54 nm and 2.05 nm for NbN films of 5 and 50 nm, respectively^18^ while the 5 nm-thick NbN films on MgO substrates exhibited *ξ*(0 K) = 4.7 nm^19^. According to the paper^20^, $\xi^{ \bot } (0{\text{ K}})$ and $\xi_{{}}^{\parallel } (0{\text{ K}})$ in NbN layers deposited on a Si/SiO_2_ substrate are equal to 3.4 and 2.6 nm for 50 nm and 2.9 and 2.3 nm for 100 nm thick film^20^. Note that the same authors^20^ got $\xi^{ \bot } (0{\text{ K}}) = 9.3{\text{ nm}}$ and $\xi_{{}}^{\parallel } (0{\text{ K}}) = 7.5{\text{ nm}}$ for Nb films with a thickness of 40 nm. Comparison of the data from different works shows that in contrast to Nb, the main superconducting parameters *λ* and *ξ* in NbN differ by nearly two orders of magnitude and 50 nm thick NbN layers are optimal for observing the expected effects, since in this case the deviation of the film thickness *d* from both parameters is about an order of magnitude: *ξ* << *d* << *λ*.

However, a small coherence length (of the order of several nanometers) together with a moderate disorder can result in the appearance of superconducting granularity with a set of regions (a few tens of nanometers in size) whose superconducting parameters slightly differ from each other. Even more, an external magnetic field enhances the inhomogeneity of the superconducting medium, as was shown in the work^21^ for NbN films, similar to ours. Conventional global transport measurements are limited in their ability to identify such regions due to the independence of sample details. That is why we used a non-local four-probe resistance measurements sensitive to inhomogeneity factors^22^.

At the end of this section, we would like to emphasize that this work has not been the first study of temperature and angular dependences of *H*_c2_ in NbN films. Next, we will refer to the earlier^23^ and recent publications^20,24^ on this subject, explain the difference between the samples and suggest explanation of the surprising 2D dimensionality of our comparatively thick NbN layers studied. We believe that the results of the work provide new insights into the two-dimensional physics of type-II superconducting films and their practical applications as the main elements of superconducting integrated circuits.

## Results

### Expected angular dependence of the upper critical field in 2D and 3D regimes

Our approach to the analysis of superconductor dimensionality is based on the different angular dependences *H*_c2_(*θ*) in 2D and 3D superconducting layers (*θ* is the angle between the magnetic field and the normal to the film), and the difference in dimensionality is conventionally understood as *d* << *ξ*, *λ* and *d* >> *ξ*, *λ*, respectively*.* The upper critical field for a certain angle *θ* can be found by knowing the out-of-plane critical field $H_{{{\text{c2}}}}^{ \bot } = H_{{{\text{c2}}}}^{{}} (\theta = 0^{0} )$ and the ratio $\gamma_{0} = H_{{{\text{c2}}}}^{\parallel } /H_{{{\text{c2}}}}^{ \bot }$ with $H_{{{\text{c2}}}}^{\parallel } = H_{{{\text{c2}}}}^{{}} (\theta = 90^{o} )$, the in-plane critical field usually strongly exceeding $H_{{{\text{c2}}}}^{ \bot }$ in 2D samples while in isotropic superconductors the two limit values coincide^7^. As follows from the analysis^7^, for 2D films we have the following relation for an anisotropy factor $\gamma (\theta ) = H_{{{\text{c2}}}}^{{}} (\theta )/H_{{{\text{c2}}}}^{{}} (0^{{\text{o}}} )$:

3 $$\left| {\gamma (\theta )\cos \theta } \right| + \left( {\gamma (\theta )\sin \theta /\gamma_{0} } \right)^{2} = 1,$$

while in a 3D anisotropic superconductor, an implicit equation that allows to find the angular dependence *H*_c2_(*θ*) looks as^25^

4 $$(\gamma (\theta )\cos \theta )^{2} + \left( {\gamma (\theta )\sin \theta /\gamma_{0} } \right)^{2} = 1.$$

**Figure 1:**
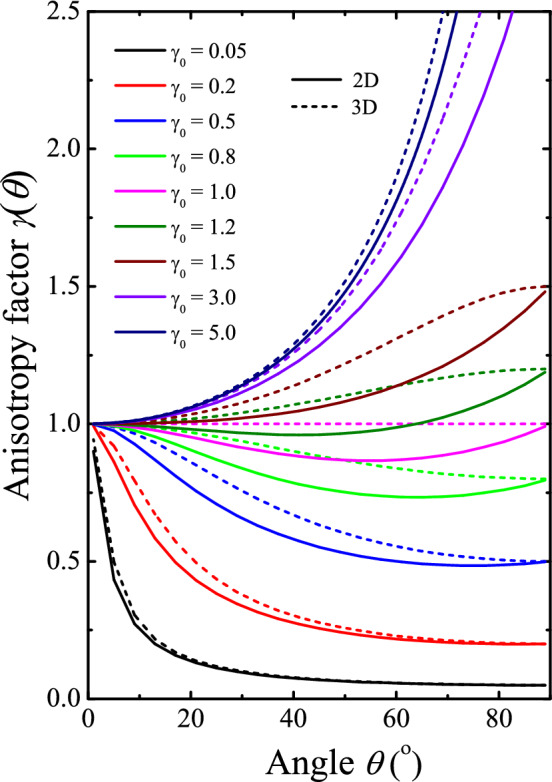
Comparison of calculated 2D and 3D angle-dependent upper critical fields normalized to the perpendicular field value *H*_c2_(*θ*)/*H*_c2_(0°). 2D (solid) and 3D (dashed) curves for different *γ*_0_ values were calculated using Eqs. (3) and (4), respectively.

Figure 1 demonstrates calculated *γ*(*θ*) dependencies for different *γ*_0_ ratios in 2D and 3D limits. Let us pay attention to the fact that the curves for both regimes are quite close to each other (at least in shape) if anisotropy is high, while a principal difference appears when the two values $H_{{{\text{c2}}}}^{\parallel }$ and $H_{{{\text{c2}}}}^{ \bot }$ are close in magnitude. It manifests itself in a wide dip seen *only* in the 2D $\gamma (\theta )$ curves for angles intermediate between 0 and 90 degrees. Therefore, our experimental task was to fabricate NbN films with anisotropy close to unity and measure the $\gamma (\theta )$ dependence for samples with various thicknesses.

Early experiments^23^ showed that depending on growth conditions, superconducting NbN films prepared by sputtering technique exhibit two types of the microstructure, that with columns normal to the film surface and voids between columns extending through the film thickness and a continuous polycrystalline structure formed by randomly oriented grains. Comparatively small difference between parallel and perpendicular critical fields was observed for the latter NbN samples^23^. The layers with column structures demonstrated enhanced critical fields $H_{{{\text{c2}}}}^{ \bot }$ as was confirmed recently^24^. Since our goal was to create films with a relatively small variations in critical magnetic fields for different orientations, we have followed the technology leading to the fabrication of polycrystalline samples^23^ that allowed us to get high-quality NbN thin layers with equiaxed grain microstructure^26^ (cf. “Methods” section).

### Non-local transport characteristics of NbN films in magnetic fields of various magnitudes and orientations

**Figure 2:**
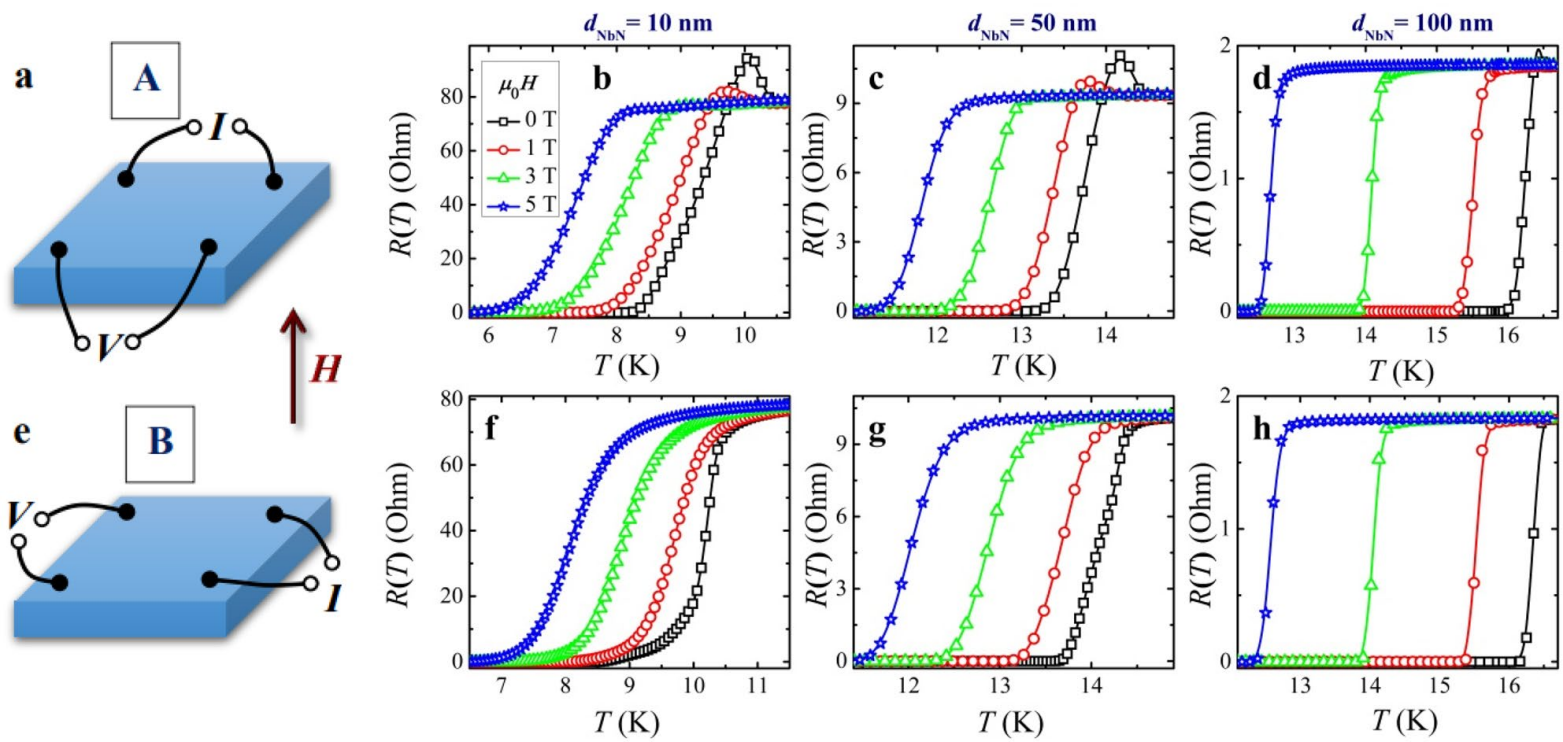
Representative four-probe resistance-versus-temperature curves *R*_A_(*T*) and *R*_B_(*T*) measured for two different non-local contact arrangements A (**a**) and B (**e**) on a square NbN superconducting layer; magnetic fields were directed normally to the sample, their values are shown in the figures. (**b**,** f**), (**c**, **g**), and (**d**, **h**) are experimental data for the NbN samples with a thickness of 10, 50, and 100 nm, respectively.

**Figure 3:**
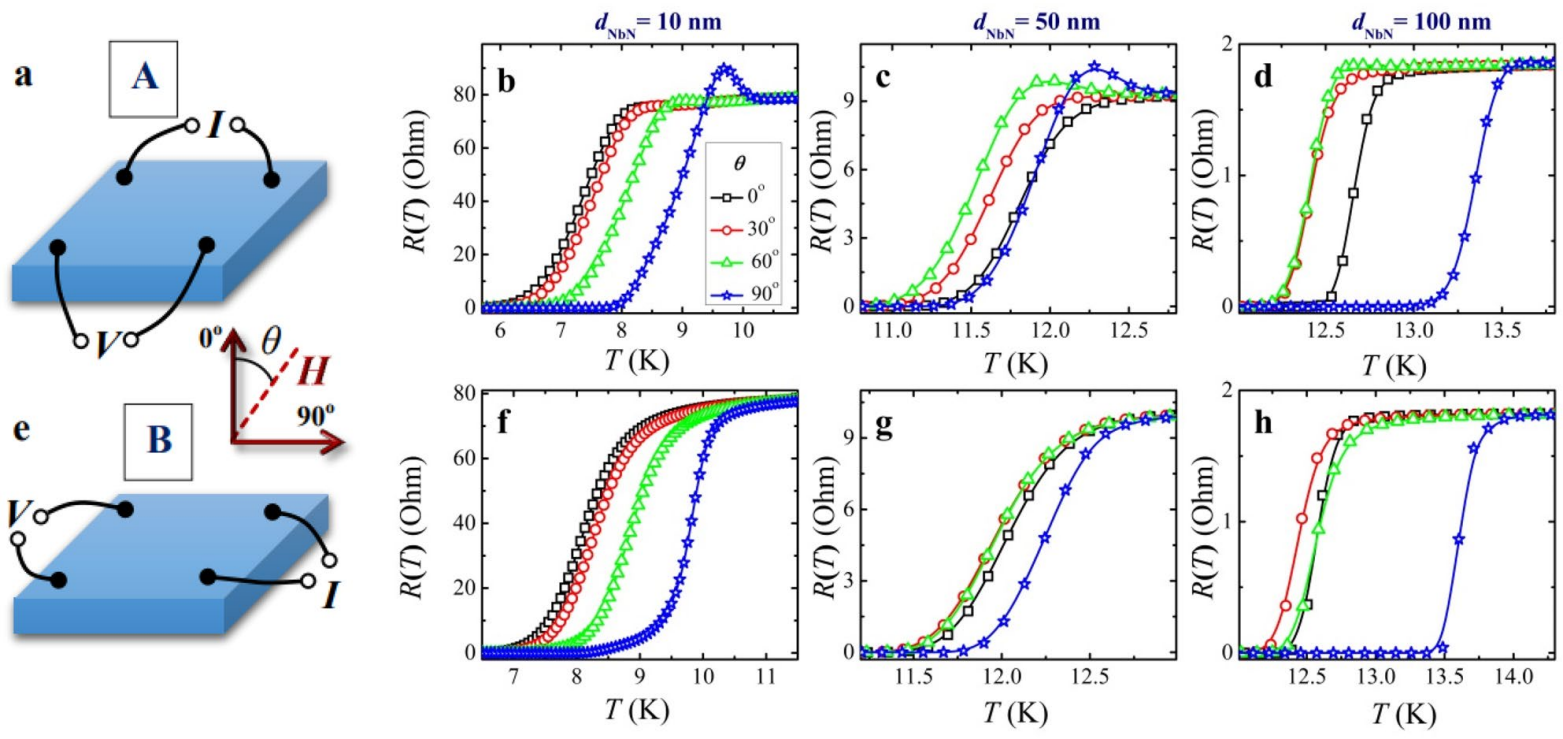
Representative four-probe resistance-versus-temperature curves *R*_A_(*T*) and *R*_B_(*T*) measured for two different non-local contact arrangements A (**a**) and B (**e**) on a square NbN superconducting layer; the orientation of the magnetic field *μ*_0_*H* = 5 T is set by the angle *θ* between the normal to the sample and the field direction, *θ* values are shown in the figures. (**b**, **f**), (**c**, **g**), and (**d**, **h**) are experimental data for the NbN samples with a thickness of 10, 50, and 100 nm, respectively.

Electrical measurements were carried out in a four-point van der Pauw configuration for temperatures near the transition from the normal to the superconducting state. In the van der Pauw approach to square films, four probes are located at the corners, in contrast to the traditional linear layout. This allows to get an average resistivity of the sample, whereas a linear array provides the resistivity only in the probing direction, see more discussion and related references in the “Supplementary information” section. Within such non-local approach^22^ described in the “Methods” section, the four-probe resistance *R* is defined as the ratio of the measured potential drop between the voltage-sensing electrodes and the current applied to other two contacts. Representative *R-vs-T* characteristics for magnetic fields of a fixed spatial orientation but varying magnitude (Fig. 2) and those of a fixed value but different orientations (Fig. 3) are shown below. The original four-probe resistance versus temperature plots, measured in magnetic fields from zero to 6 T in 0.5 T increments, are presented in the “Supplementary information” section. In Figs. 2 and 3 we demonstrate our resistance data for some selected fields and their orientations.

**Figure 4:**
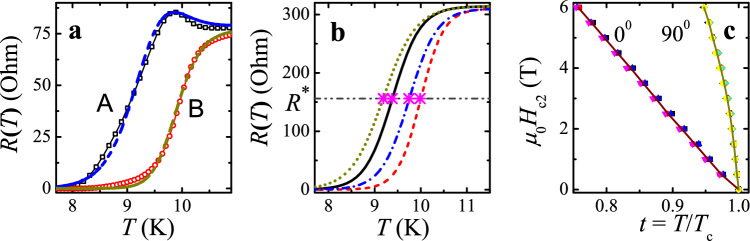
Illustration of the non-local four-probe method for determining the temperature dependence of the upper critical magnetic film *H*_c2_, a non-local resistance-vs-temperature characteristics of a 10-nm thick NbN film measured for two configurations A and B shown in Figs. 1 and 2 in a perpendicular magnetic field *μ*_0_*H* = 0.5 T, **b** restored *R*_*i*_(*T*) traces for four different sections of the sample (solid, dashed, dotted and dashed-dotted curves correspond to *i* = 1, 2, 3, 4, respectively), the crosses indicate positions of middle points in the transition curves, *R*_*i*_(*T*_c,*i*_)** = **R*, cf. “Methods” section, **c** comparison of the *H*_c2_-vs-*t* curves for parallel $(\theta = 90^{ \circ } )$ and perpendicular $(\theta = 0^{ \circ } )$ magnetic fields found by using the four *R*(*T*) traces (symbols) and the averaged over them curve (lines).

In samples with superconducting granularity like those in the work^21^ and ours, different sections of the film can have slightly different temperatures *T*_c_ and transition widths Δ*T*_c_. Because *R*(*T*) curves obtained by non-local four-probe resistive measurements are a combination of corresponding characteristics in four individual sections of the sample, their direct interpretation (e.g., for estimating *T*_c_) is impossible and require preliminary processing of the measured data, the results of which are given below. This conclusion applies even to a qualitative analysis of the obtained *R*_A_(*T*) and *R*_B_(*T*) dependencies in Figs. 2 and 3. As was explained in our recent paper^22^ (cf. also “Methods” section), occasionally, it can lead to spurious features as near-*T*_c_ resistance peaks seen in some curves in Figs. 2 and 3. Such feature is actually a manifestation of small *T*_c_ variations in adjacent sections of the film and disappear in the reconstructed *R-*vs*-T* curves for four section resistances between two adjacent contacts^22^. Of course, such a four-probe configuration significantly complicates the analysis of the resistive measurements. Nevertheless, the approach makes it possible to compare the resistance behavior in different fragments of the film and reveal more reliably its specific features, which are not local properties of the samples, but rather their universal characteristics, see Fig. 4.

Using measured *R*_A_(*T*) and *R*_B_(*T*) curves for a 10 nm thick NbN film in a perpendicular magnetic field *μ*_0_H = 0.5 T as an example (see Fig. S2 in the “Supplementary information” section), we demonstrate in Fig. 4 the origin of anomalies in non-local four-probe *R*(*T*) curves as well as the ability of finding corresponding characteristics for four different sections of the film simultaneously. It is clear that the observed appearance (or in some cases, disappearance) of resistance peaks with a change in the external parameters as in Fig. 4a is caused by the play of parameters, and do not have any principal significance. Using related formulas from the paper^22^ (cf. also “Methods” and “Supplementary information” sections), we found separate *R*_*i*_(*T*) resistances of the four sections (Fig. 4b) which together form the temperature dependences *R*_A_(*T*) and *R*_B_(*T*) of the four-contact resistances shown in Fig. 4a. What is most important is that the *R*_*i*_(*T*) behavior for separate film parts with slightly different critical temperatures *T*_c,*i*_ is fundamentally similar as can be clearly seen from Fig. 4b. Even more, we have found that in the vast majority of cases, the *H*_c2_-vs-temperature dependences for the four different sections of the film practically coincided when they were plotted as functions of the ratio *t* = *T*/*T*_c_ rather than the temperature itself, see Fig. 4c. This finding considerably increases the reliability of our conclusions and allows us to demonstrate *H*_c2_(*t*) and *H*_c2_(*θ*) curves obtained from averaged over the four sections *R*(*T*) characteristics (the lines in Fig. 4c). Note a strong difference between the two limit values of the upper critical field in the ultra-thin NbN film.

We want again to draw attention to the fact already stressed in our previous work^22^ that the appearance of a surprising near-*T*_c_ resistance peak in this case is an artifact that has no physical meaning and is arising due to the specific arrangement of contacts in non-local four-probe measurements and the presence of a slight difference in *T*_c_’s for adjacent areas of the sample^22^. As can be seen from Fig. 4b, there is no such feature in any part of the film, where the *R*(*T*) traces are *monotonically* decreasing curves in the narrow region of the normal-to-superconducting state transition, as one would expect. Notice that when the *R*(*T*) dependence is measured using an in-line configuration when the voltage probes are located between the current contacts, the near-*T*_c_ resistive peak disappears, see Fig. 4 in our paper^22^.

### Temperature dependencies of upper critical magnetic fields in NbN films of various thicknesses

**Figure 5:**
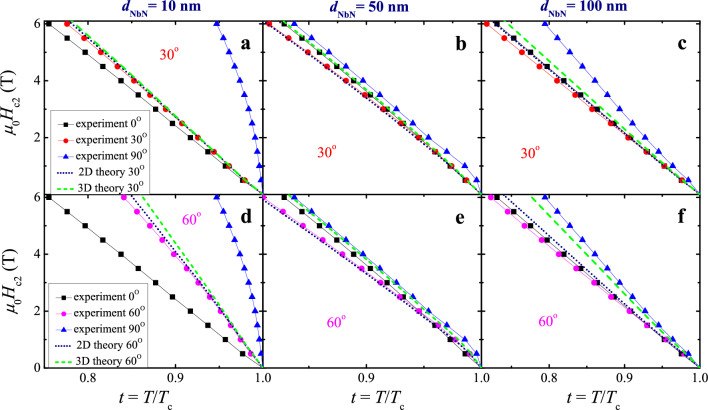
Representative *H*_c2_-vs-*T/T*_c_ dependencies for three 10, 50, and 100 nm thick NbN films with different orientations (symbols), the lines provide guides to the eye. Experimental data are compared with related calculations for 2D [Eq. (3), dotted lines] and 3D [Eq. (4), dashed lines] regimes.

Figure 5 demonstrates the temperature dependencies of $H_{{{\text{c2}}}}^{ \bot }$ and $H_{{{\text{c2}}}}^{\parallel }$ critical fields thus obtained. For the three studied thicknesses, $H_{{{\text{c2}}}}^{ \bot } (T)$ is a linear function of temperature excluding a very narrow region near *T*_c_. This indicates a clear 3D behavior and allows us to estimate related zero-temperature values by extrapolating measured data to *T* = 0. As a result, using the 3D formula (1) we get $\mu_{0} H_{{{\text{c2}}}}^{ \bot } (0{\text{ K}})$ = 25, 35, 23 T and $\xi_{{}}^{\parallel } (0{\text{ K}})$ = 3.6, 3.1, 3.8 nm for *d* = 10, 50, and 100 nm. As for the parallel critical magnetic field $H_{{{\text{c2}}}}^{\parallel }$, there is the fundamental difference between samples with a thickness of 10 nm and others. In the latter case, we are again dealing with the 3D scenario and the dependence $H_{{{\text{c2}}}}^{\parallel } (T)$ remains linear, as for the perpendicular field $H_{{{\text{c2}}}}^{ \bot } (T)$. Extrapolation to zero temperature gives $\mu_{0} H_{{{\text{c2}}}}^{\parallel } (0{\text{ K}})$ = 37, 28 T and $\xi_{{}}^{ \bot } (0{\text{ K}})$ = 2.9, 3.1 nm for *d* = 50 and 100 nm, respectively. These estimates reasonably agree with most literature results (cf. “Introduction” section). At the same time, the temperature dependence $H_{{{\text{c2}}}}^{\parallel } (T)$ for a 10 nm-thick sample clearly deviates from linear behavior, as should be for a film whose thickness is comparable to the coherence length. Using the two-dimensional square-root behavior following from Eq. (2), we find that $\mu_{0} H_{{{\text{c2}}}}^{\parallel } (0{\text{ K}})$ ≈ 31 T for *d* = 10 nm, which gives us, from the same relation, $\xi_{{}}^{\parallel } (0{\text{ K}})$ = 3.7 nm agreeing well with the 3.6 nm estimate derived above from the $\mu_{0} H_{{{\text{c2}}}}^{ \bot } (0{\text{ K}})$ value.

**Figure 6:**
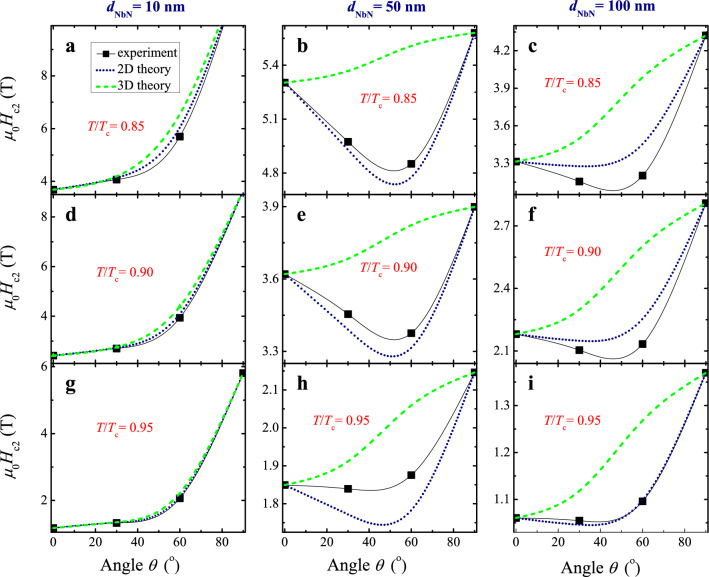
Representative *H*_c2_-vs-*θ* dependencies for three 10, 50, and 100 nm thick NbN films measured at three near-*T*_c_ temperatures in magnetic fields of different orientations (squares), the black lines provide guides to the eye. Experimental data are compared with related calculations for 2D [Eq. (3), dotted lines] and 3D [Eq. (4), dashed lines] regimes. *H*_c2_ values for *θ* = 90° are not shown in (**a**) and (**d**) since they were outside the magnetic fields available to us and, therefore, were not measured.

It turned out to be much more interesting to compare the temperature dependences $H_{{{\text{c2}}}}^{{}} (T)$ measured for *θ* = 30° and 60°, with those expected theoretically from relations (3) and (4) using *γ*_0_(*T*) values taken from the experiment. It follows from Fig. 5 that for *all* three thicknesses the 2D approximation describes experimental results better than that for 3D superconductors. Whereas for samples with a thickness of 10 nm such behavior could be anticipated, since the thickness value *d* is comparable to the coherence length, a similar result for much thicker NbN layers is indeed unexpected. However, since the difference of 2D and 3D behaviors does not seem sufficient to make a convincing conclusion, we needed additional experiments where the dissimilarity between the 2D and 3D curves would be so obvious that the preference for one of the two approaches (3) or (4) would be self-evident. We did it by comparing the measured and theoretically expected angular dependences *H*_c2_(*θ*) for different *t* = *T/T*_c_ ratios (Fig. 6).

### Angular dependencies of upper critical magnetic fields in NbN films of various thicknesses

To find the expected *H*_c2_-vs-*θ* dependences for different *t* = *T/T*_c_ ratios (Fig. 6), we followed the same procedure that was used above for the calculations of *H*_c2_-vs-*T/T*_c_ characteristics shown in Fig. 5. Corresponding traces for magnetic fields directed normally $H_{{{\text{c2}}}}^{ \bot } = H_{c2} (\theta = 0^{ \circ } )$ and parallel $H_{{{\text{c2}}}}^{\parallel } = H_{{{\text{c2}}}}^{{}} (\theta = 90^{ \circ } )$ to the NbN films were taken as initial ones, which were substituted into Eqs. (3) and (4) to find predicted critical field *H*_c2_(*θ*) as a function of the angle *θ* and compare it with the measured one. Again, the experimental data for a 10 nm-thick film was better described by the 2D formula (3) which well approximates the behavior in general although some deviations of the experimental values from expected ones was observed at *T* < 0.95*T*_c_. However, the most striking result was that for the thicker samples, when the agreement with the 2D formula (3) was even more convincing, including a dip in the angular dependence of *H*_c2_ at intermediate angles *θ* between 0 and 90° predicted by the 2D regime as opposed to the 3D case, see Fig. 1. In particular, there is an almost perfect agreement between calculations by Eq. (3) and the experimental data for films 50 nm thick at *T* < 0.95*T*_c_ and 100 nm thick at *T* > 0.9*T*_c_. In the next section, we will explain this seeming contradiction and discuss its importance for practical applications of the NbN films.

## Discussion

Before comparing magneto-transport characteristics of NbN films with three different thicknesses, we should realize the reason for the rather large difference in their critical temperatures and find out whether it can significantly affect the angular dependence of their critical fields. As was argued in Ref.^27^, the main factor influencing *T*_c_ evolution in the NbN system is disorder that can be characterized by a single value *k*_F_*l* known as the Ioffe–Regel parameter that is a measure of the electronic mean free path *l* in units of de-Broglie wavelength, *k*_F_ is the Fermi wave vector. Using the phase *T*_c_-vs-*k*_F_*l* diagram (Fig. 7 in Ref.^27^), we find that *k*_F_*l* is approximately 4, 6 and 7 for films with thicknesses of 10, 50 and 100 nm, respectively. It means that the three samples belong to the moderate disorder limit (4 < *k*_F_*l* < 10), when the system follows the mean field Bardeen-Cooper-Schrieffer behavior, with the superconducting energy gap vanishing at the temperature at which electrical resistance appears^27^.

To find the true cause of the unusual *H*_c2_-vs-*θ* behavior for 50 and 100 nm thick NbN films, let us turn to the analysis of related arguments^7^ leading to simple analytical relations (3) and (4) which are actually interpolation formulas between parallel $H_{{{\text{c2}}}}^{\parallel } = H_{{{\text{c2}}}}^{{}} (\theta = 90^{ \circ } )$ and perpendicular $H_{{{\text{c2}}}}^{ \bot } = H_{{{\text{c2}}}}^{{}} (\theta = 0^{ \circ } )$ critical fields. In an anisotropic 3D case, the two components of the magnetic field caused by the field penetration into the sample through quantized vortices are additive terms in the energy density which are proportional to $H_{{{\text{c2}}}}^{\parallel 2}$ and $H_{{{\text{c2}}}}^{ \bot 2}$, respectively. As a result, their sum has a simple ellipsoidal form (4)^25^. Close enough to *T*_c_, $\xi_{{}}^{ \bot } (T)$ is always large to justify the 2D approximation but away from *T*_c_, the temperature-dependent coherence length shrinks below the thickness *d* and at $T \le 0.95T_{{\text{c}}}$, $\xi_{{}}^{ \bot } (T)$ is already much less than *d* in 50 and 100 nm thick films. It means that from the point of view of the spatial distribution of the superconducting order parameter, we are dealing with a 3D continuum in which the coherence length is almost isotropic, hence, $H_{{{\text{c2}}}}^{ \bot }$ and $H_{{{\text{c2}}}}^{\parallel }$ nearly coincide. Nevertheless, in such comparatively thick NbN layers, the* d* value remains much smaller than the magnetic penetration depth. If so, the component of the external magnetic field $H^{\parallel }$ aligned parallel to the film penetrates the entire film and induces diamagnetic supercurrents which are proportional to $H^{\parallel }$ and varying linearly with the depth^28^. It leads to a kinetic energy density term that is again proportional to $H_{{}}^{\parallel }$ squared^29^. The impact of the perpendicular component $H^{ \bot }$ is reduced to the formation of quantum vortices enclosing a magnetic flux Φ_0_: $\pi r^{2} \mu_{0} H^{ \bot } \sim \Phi_{0}$ where *r* is their characteristic linear size^29^. Due to the relationship between *r* and $H^{ \bot }$, related contribution to the total kinetic energy density is proportional to $H^{ \bot }$ (instead of $H^{ \bot }$ squared) that after normalizing by $H_{{{\text{c2}}}}^{ \bot }$ and $H_{{{\text{c2}}}}^{\parallel }$ leads to Eq. (3) ^7^.

The validity of the statement about the 2D-like spatial distribution of the magnetic field in the relatively thick NbN films is a consequence of the specific values of the penetration depth. We can estimate it using the weak-coupled BCS expression for the Ginzburg–Landau penetration depth^30^ $\lambda_{{{\text{GL}}}} (0) = 64\left[ {\rho (\mu \Omega \;{\text{cm)/}}T_{{\text{c}}} ({\text{K}})} \right]^{1/2} ({\text{nm}})$. The resistances of 10-, 50-, and 100-nm thick layers, estimated in the “Methods” section, were 360, 210, and 80 μΩ cm, hence, the penetration depths *λ*_GL_(0) are estimated at 430, 250 and 140 nm, respectively. In fact, this magnitude is underestimated because in strongly-coupled superconductors, such as niobium nitride, there is an additional factor significantly increasing the value of *λ*^30,31^.

Very good agreement with Eq. (4) for 50- and 100-nm thick NbN films means that orbital effects, rather than the spin response, predominate the upper critical fields in relatively thick NbN layers. It contrasts, in particular, with related conclusions for nickelates where strongly Pauli-limited superconductivity takes place and, as a result, the upper critical field value is almost isotropic^32^ in spite of the same layered crystal structure as in infinite-layer cuprates.

The goal of the work was to demonstrate the possibility of realizing in a superconducting film a state, which is three-dimensional from the viewpoint of the superconducting order parameter and two-dimensional relating supercurrent spatial distribution. To do this, the thickness of the layer should be somewhere between the two characteristic lengths, *ξ* and *λ*, which in turn have to differ radically in magnitude. An ideal solution is NbN films with a thickness of 50–100 nm characterized by nearly isotropic superconducting state, a sign of a 3D nature, and, at the same time, a fairly uniform dissipationless current as in very thin 2D superconducting films.

This property, a new advantageous feature of niobium nitride films in addition to the already known^9^, can be useful for the employment of relatively thick NbN layers in superconducting integrated circuits. Indeed, interest in developing superconductor digital electronics using Josephson junctions has been recently renewed due to a search for energy saving solutions in applications related to high-performance computing^33^. However, Josephson junctions currently occupy less than eleven percent of the circuit area, with inductors and flux transformers taking up the remainder^34^. To solve this problem, a material with a relatively high kinetic inductance is needed, instead of the conventional niobium. This can be achieved using a superconductor, in which the magnetic penetration depth is much greater than the film thickness and the supercurrent is flowing uniformly through it^34^. As stated in Ref.^34^, it is convenient to use transition metal nitrides for this purpose. The above results testify in favor of niobium nitride films several tens of nanometers thick at temperatures well below *T*_c_ when the NbN film is definitely three-dimensional from the viewpoint of the order parameter. This is quite suitable for large-scale computing systems supposed to operate at a temperature of 4.2 K^35^. Appropriate measures to control NbN layer parameters are essential to find optimal fabrication conditions in order to realize an isotropic superconducting layer with uniform supercurrent across it.

## Methods

### Sample preparation

NbN films of thicknesses from 10 to 100 nm were deposited by pulsed laser deposition on *c*-cut Al_2_O_3_ substrates kept at a constant temperature of 600 °C. After deposition, chemical and structural properties of the NbN layers were characterized by several analytical techniques, see the details in Refs.^26,36^ and in the “Supplementary information” section.

The samples had a square shape with contacts at the corners. Such contact arrangement made it possible to carry out non-local four-probe measurements which, as was argued by us in Ref.^22^, strongly enhance sensitivity to inhomogeneity factors and make related experiments on superconducting films the method of choice for knowing the spatial distribution of a superconducting order parameter and at the same time to get more reliable information about universal transport features, which are not dependent on the local properties of the samples. The resistivity of NbN layers typically measured at 20 K *ρ*_20_ significantly depends on the type of the substrate, see Table 2 in our work^36^ and the film thickness *d*.

Experimental plots presented in Figs. 2 and 3 as well as in the “Supplementary information” section show almost identical normal-state resistances in A and B configurations: *R*_A_ ≈ *R*_B_. If so, then we can use a simplified version of the Van der Pauw's formula for electrical resistivity *ρ* = π*dR*_A_/ln2^37^. For NbN films of thicknesses 10, 50, and 100 nm we obtain the following values—360, 210, and 80 μΩ cm, respectively. The decrease in resistivity with increasing thickness *d* can be qualitatively explained by reduced contribution from electron scatterings at the thin-film boundaries^38^ that is quantitatively described by the relation^39^ *ρ* = const·(1 + 3* l*/8*d*) with *l*, the electron mean free path.

### Electrical measurements

We have measured *R*(*T*) dependences for two combinations of current-carrying and voltage contacts (A and B in Figs. 2 and 3) the knowledge of which makes it possible to find the temperature dependence of the four section resistances between two adjacent contacts, see Ref.^22^. The measurements were performed by the Physical Property Measurements System (PPMS) DynaCool (Quantum Design). Additional experimental data of *R*(*T*) measurements are shown in the “Supplementary information” section (see Figs. S2–S4). In the “Supplementary information” section we explain the relationship of our approach to the well-known van der Pauw method, which uses four contacts placed around the perimeter of the sample and thus gives its average resistance, in contrast to the linear four-point method that provides knowledge of resistivity only in the probing direction.

### Extraction of the local resistance-versus-temperature traces from non-local four-probe measurements

Following Ref.^22^, the non-local four-probe measurements performed for two configurations A and B shown in Figs. 2 and 3 were analyzed using the oversimplified resistive model where the superconducting film was conditionally divided into four resistive regions, see below two equivalent circuits with four resistances of the same normal-state value* R.* The derivation of the main equation for a non-local four-probe resistance can be found in the “Supplementary information” section.
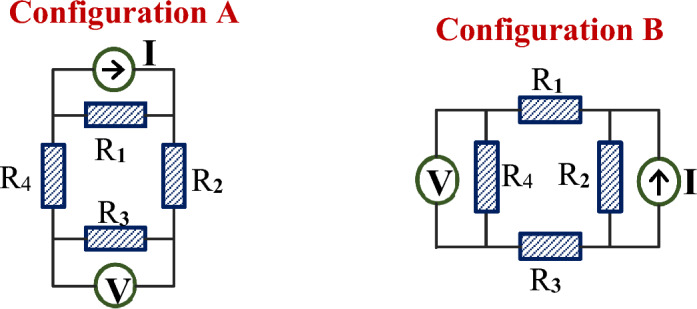


Applying Kirchhoff's laws to the electric circuits above, we obtain resistance values measured by the four-contact method: $R_{{\text{A}}} (T) = \frac{{R_{1} (T) \cdot R_{3} (T)}}{{R_{1} (T) + R_{2} (T) + R_{3} (T) + R_{4} (T)}}$ and $R_{{\text{B}}} (T) = \frac{{R_{2} (T) \cdot R_{4} (T)}}{{R_{1} (T) + R_{2} (T) + R_{3} (T) + R_{4} (T)}}$ where *R*_*i*_(*T*) (*i* = 1, 2, 3, 4) are resistance-vs-temperature dependencies for each section of the film (see the “Supplementary information”) which are approximated by identical formulas $R(T) = R^{*}\cdot \left( {1 + \tanh \left( {(T - T_{{\text{c}}} )/\Delta T_{{\text{c}}} } \right)} \right)$ with $R_{{}}^{ * } = R(T_{{\text{c}}} )$, the midpoint of the resistance drop. Therefore,* T*_c_ is identified as the temperature at which the resistive transition reaches 50% of the normal-state value. An example of four separate *R*-vs-*T* traces which together form the temperature dependences *R*_A_(*T*) and *R*_B_(*T*) in Fig. 4a is shown in Fig. 4b where the crosses indicate positions of *middle points* in the *R*_*i*_(*T*) transition curves. Their positions along the temperature axis are identified as *T*_c_ values for a given area of the sample. Next, we have used the averaged over the four sections *R*(*t* = *T*/*T*_c_) characteristics for deriving *H*_c2_(*t*) and *H*_c2_(*θ*) curves as discussed below.

### Extraction of the upper critical magnetic field values from resistive characteristics

The simplest way to find the $H_{{{\text{c2}}}} (T)$ dependence for type-II superconducting condensates is resistive *R*(*T*) measurements across the normal metal-to-superconductor transition fixed by the resistance drop. In this case, the transition temperature *T*_c_(*H*) in an applied magnetic field *H* measures the upper critical field as *H*_c2_(*T* = *T*_c_(*H*)) = *H.* Related temperature and angular dependencies of the upper critical field *H*_c2_ are shown in Figs. 2 and 3.

### Supplementary Information

Supplementary Information.

## Data Availability

The datasets generated during and/or analyzed during the current study are available from the corresponding author on reasonable request. Besides it, the detailed information about the main characteristics of 50 nm thick NbN layers can be found at https://doi.org/10.2478/jee-2019-0047. Source dataset for the three types of NbN films served as an input for the outcomes shown in Figs. 5 and 6 is presented in the “Supplementary information” section.
