# Supplementary material for: Competing length scales and 2D versus 3D dimensionality in relatively thick superconducting NbN films
Source: Sci Rep. 2023 Nov 9;13:19450. doi: 10.1038/s41598-023-46579-x (PMC10636059; doi:10.1038/s41598-023-46579-x)
Supplement: Supplementary file 1 — Supplementary Information. [file 41598_2023_46579_MOESM1_ESM.pdf]

Supplementary information for

Competing length scales and 2D versus 3D dimensionality in relatively thick superconducting NbN films

Mikhail Belogolovskii*, Magdaléna Poláčková, Elena Zhitlukhina, Branislav Grančič, Leonid Satrapinskyy, Maroš Gregor, Tomáš Plecenik

*belogolovskii@ukr.net

NbN film fabrication and characterization

The pulsed laser deposition of NbN films was carried out in an ultrahigh vacuum chamber using an excimer KrF laser with the 248 nm wavelength, the pulse duration of 35 ns, and the laser fluency of 4.94 J·cm^{-2}. The NbN thin films were deposited on *c*-cut Al_2O_3 substrates, ultrasonically cleaned in acetone, isopropanol and deionized water, from a 2-inch Nb target (99.9%) in the N_2 + 1% H_2 reactive atmosphere at the gas flow of 80 sccm and the pressure of 9.3 Pa. The substrate temperature was kept constant at 600 ^{o}C.

After deposition, the NbN thin films were characterized in detail by several analytical techniques including X-ray diffraction, X-ray reflectivity, scanning electron microscopy, energy-dispersive X-ray spectroscopy and atomic force microscopy. A complete set of characterization results for the most studied 50-nm thick layers can be found in Refs. S1 and S2. Below we compare $\theta/2\theta$ XRD patterns for three NbN films with thicknesses of 10, 50, and 100 nm whose magneto-transport properties are analyzed in the work. In all three samples, we see a very strong (111) preferential orientation of fcc-NbN on *c*-cut Al_2O_3 substrate. The XRD spectra also show side-oscillations, especially clearly visible on the 10 nm thick sample due to perfect lattice coherency throughout its thickness. The period of oscillations corresponds to the thickness of each film indicating a high degree of out-of-plane crystallinity. In general, the crystal structure appears to be identical, although some subtle details require further study.

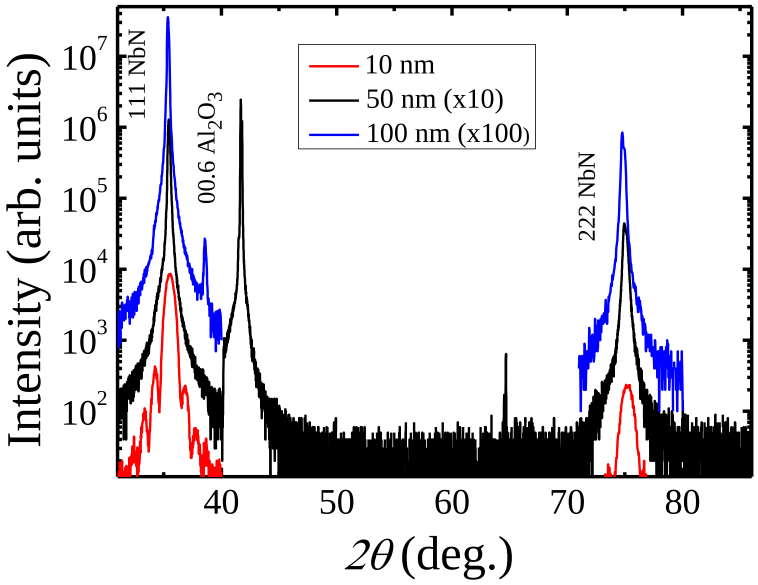

Figure S1. Three symmetric $\theta/2\theta$ XRD patterns for NbN films on *c*-cut Al_2O_3 substrates with thicknesses of 10 (red), 50 (black), and 100 (blue) nanometers

Non-local four-probe resistance-vs-temperature data for two configurations A and B shown in the "Methods" section, three NbN film thicknesses and four magnetic-field orientations (0^0, 30^0, 60^0, and 90^0)

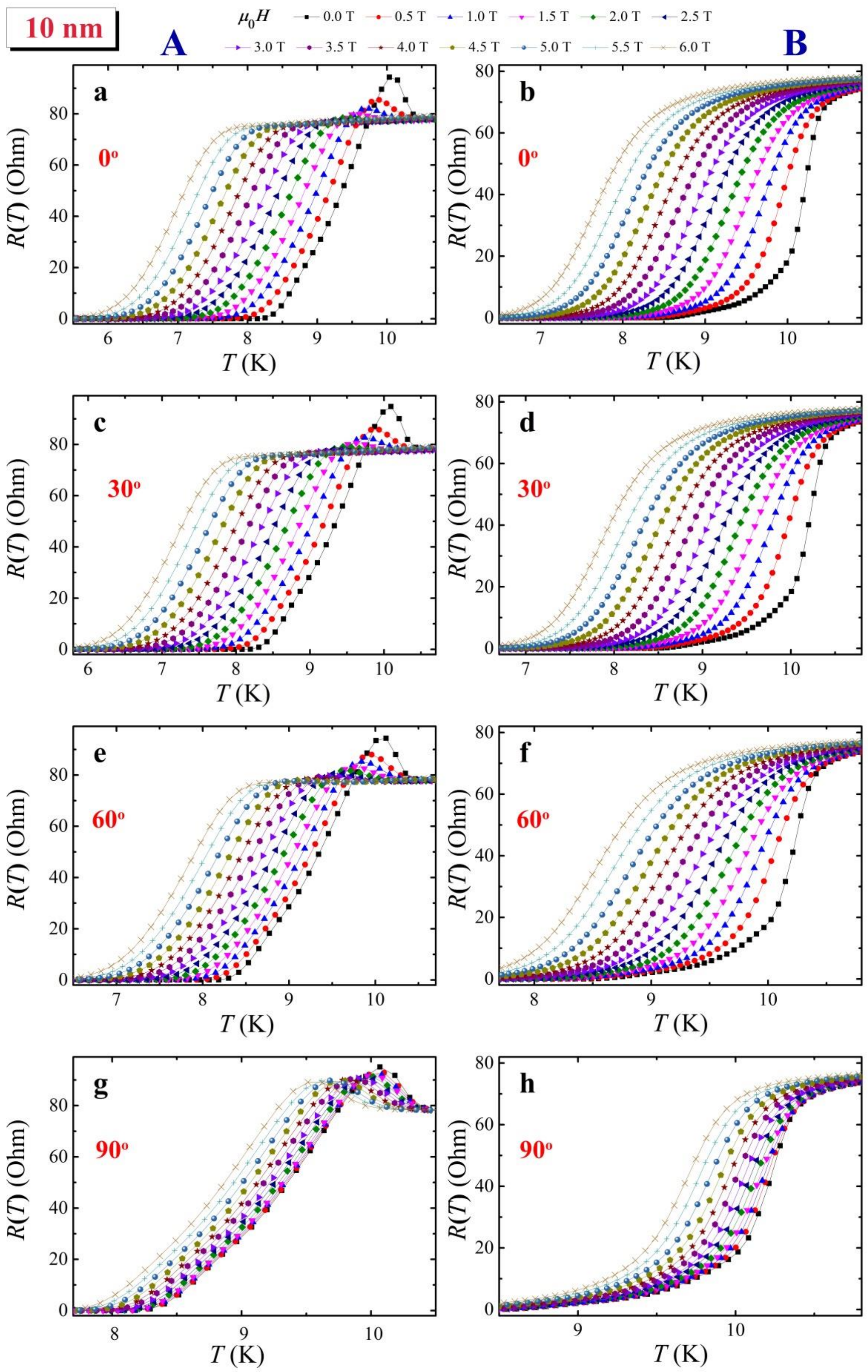

Figure S2. Four-probe resistance-vs-temperature traces for a representative 10-nm thick NbN film

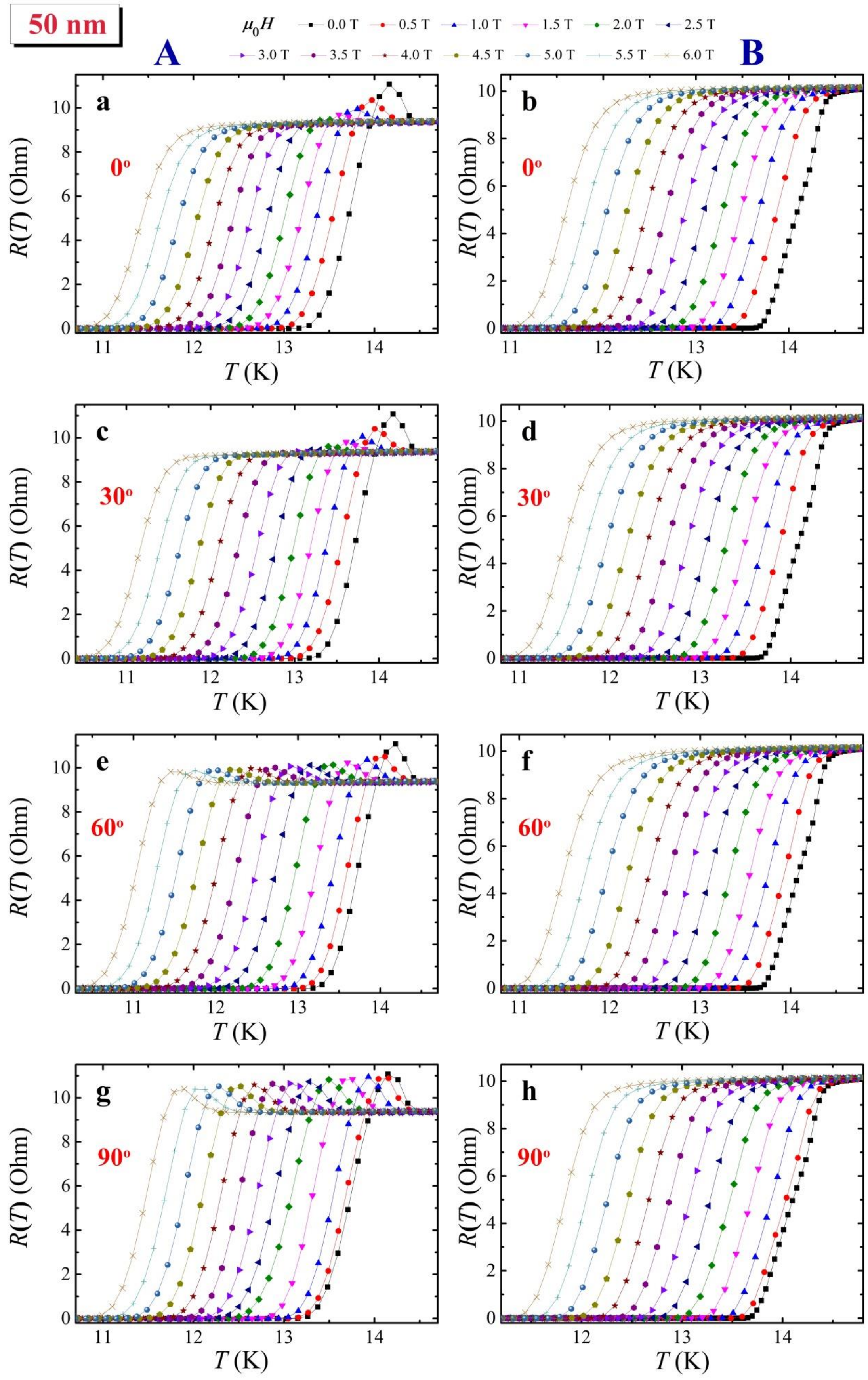

Figure S3. Four-probe resistance-vs-temperature traces for a representative 50-nm thick NbN film

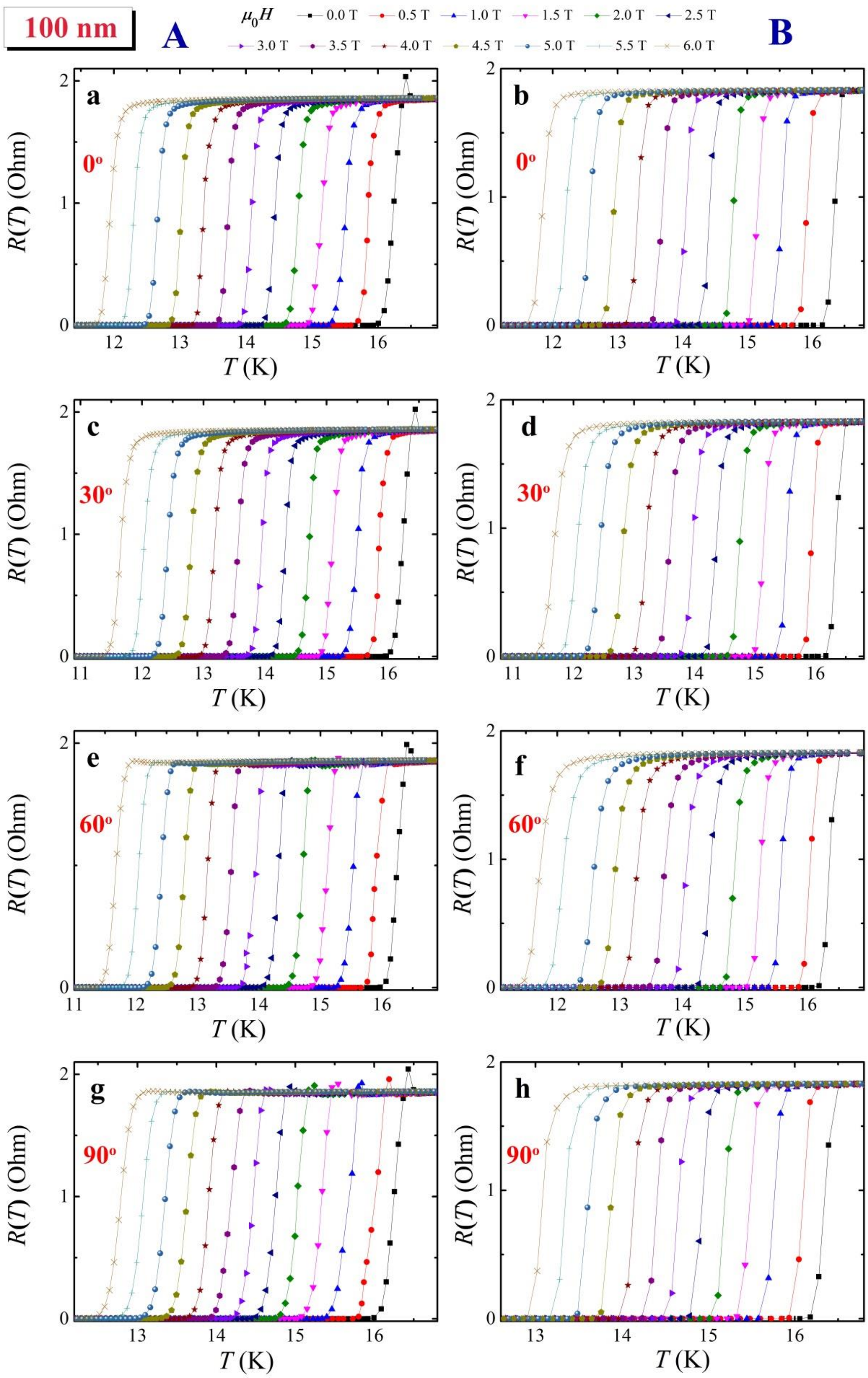

Figure S4. Four-probe resistance-vs-temperature traces for a representative 100-nm thick NbN film

Relation of our non-local four-probe approach to the van der Pauw method

Our approach to analyzing the superconducting heterogeneity in thin current-conducting films goes back to the well-known modification of the four-probe method for measuring the in-plane sheet resistance which was proposed in 1958 by van der Pauw [S3]. Solving the general problem of potential distribution in thin conductive layers of any shape and placing four probes at the film edges made it possible to carry out measurements that give a weighted average of local resistances of the sample, whereas the traditional linear layout of the four contacts provides knowledge of resistivity only in the probing direction [S4]. In the van der Pauw arrangement, a current is flowing along one edge of the sample and the voltage across the opposite edge is measured, see Fig. S5 below. From these two values, the four-probe resistance can be found using conventional Ohm's law.

However, for correct measurement, certain conditions must be met. The thickness *d* of the film, which should not have holes and/or islands of highly conductive material, has to be uniform and small in relation to the distance between the probes. And most importantly, the samples must be *homogeneous* and *isotropic*. Our control measurements of a local normal-state resistance using the standard in-line contact arrangement showed that the samples are quite homogeneous from the viewpoint of the normal state, but there is a noticeable spread in the critical temperatures and transition widths in the superconducting state. The latter circumstance has already been noted in the literature, specifically, for niobium nitride films [S5].

The authors of the work [S4] drew attention to the fact that in rather inhomogeneous samples the van der Pauw method [S3] can lead to non-physical results and, to confirm this, proposed a simplified four-resistor model. In the main text of the paper, we follow the model with a square thin layer conditionally divided into four resistive regions [S4] and consider an equivalent electrical circuit with a current source and four resistors R_i (i = 1,2,3,4).

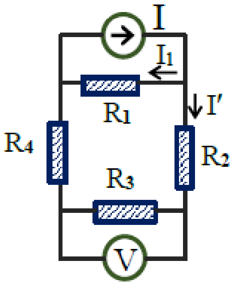

Figure S5. Equivalent four-resistor circuit for van der Pauw resistive measurements of a square superconducting layer conditionally divided into four resistive regions

First and second Kirchhoff laws allow us to obtain the following relations for currents in the circuit, see Fig. S5: $I = I_1 + I'$ and $I'(R_2 + R_3 + R_4) - I_1R_1 = 0$. Using the equations, we find that I =

$I'(R_1 + R_2 + R_3 + R_4)/R_1$. The ratio of the potential drop $V = I'R_3$ across the resistor R_3 and the current I applied has units of resistance and is known as the 'four-probe resistance' $R = V/I$ [S4, S6]. Therefore, we get [S4]

$$R = \frac{R_1 \cdot R_3}{R_1 + R_2 + R_3 + R_4}$$

In the original work [S3], van der Pauw showed that the sheet resistance of samples with arbitrary shapes can be determined from two resistance values, measured along horizontal and vertical edges, see arrangements A and B in Figs. 2 and 3. In our paper, we use the van der Pauw contact placement and four-probe resistance measurements for the two configurations in order to analyze near-T_c resistance-vs-temperature data for superconducting NbN films, see Fig. S5. As was shown in the previous publication [S7], an unusual shape of such nonlocal four-point resistance characteristics can serve as a qualitative indicator of the presence of superconducting granularity in the sample studied.

REFERENCES

S1. Volkov, S. et al. Superconducting properties of very high quality NbN thin films grown by pulsed laser deposition. *J. Electr. Eng.* **70**, 89-94 (2019). https://doi.org/10.2478/jee-2019-0047

S2. Roch, T. et al. Substrate dependent epitaxy of superconducting niobium nitride thin films grown by pulsed laser deposition. *Appl. Surf. Sci.* **551**, 149333 (2021). https://doi.org/10.1016/j.apsusc.2021.149333

S3. van der Pauw, L.J. A method of measuring the resistivity and Hall coefficient on lamellae of arbitrary shape. *Philips Tech. Rev.* **13**, 1-9 (1958).

S4. Koon, D.W. and Knickerbocker, C.J. What do you measure when you measure resistivity? *Rev. Sci. Instrum.* **63**, 207-210 (1992). https://doi.org/10.1063/1.1142958

S5. Ganguly, R. et al. Magnetic field induced emergent inhomogeneity in a superconducting film with weak and homogeneous disorder. *Phys. Rev. B* **96**, 054509 (2017). https://doi.org/10.1103/PhysRevB.96.054509

S6. Kjeldby, S.B. et al. Probing dimensionality using a simplified 4-probe method. *J. Phys.: Condens. Matter* **29**, 394008 (2017). https://doi.org/10.1088/1361-648X/aa8296

S7. Poláčková, M. et al. Probing superconducting granularity using nonlocal four-probe measurements. *Eur. Phys. J. Plus* **138**, 486 (2023). https://doi.org/10.1140/epjp/s13360-023-04123-w
